# Supplementation of EPA and DHA in pregnant women with type 1 diabetes mellitus

**DOI:** 10.1080/07853890.2021.1936151

**Published:** 2021-07-01

**Authors:** Marina Ivanisevic, Marina Horvaticek, Karlo Delmis, Josip Delmis

**Affiliations:** a Referral Center for Diabetes in Pregnancy, Ministry of Health Republic of Croatia, Clinical Department of Obstetrics and Gynecology, Zagreb University Hospital Center, School of Medicine, University of Zagreb, Zagreb, Croatia; b Institute Rudjer Boskovic, Zagreb, Croatia; c Montanuniversitat Leoben, Loeben, Austria

**Keywords:** Diabetes mellitus type 1, pregnancy, supplementation, EPA, DHA, fatty acids, omega-3, omega-6, macrosomia

## Abstract

**Background/objective:**

Lower proportions of n-3 PUFAs have been observed in neonates born to diabetic mothers. We aimed to investigate the association between DHA and EPA supplementation during pregnancy complicated with type 1 diabetes on concentration and proportion of fatty acids in maternal and foetal blood.

**Subjects and methods:**

We conducted a prospective randomized, single-blinded, placebo-controlled trial of 111 eligible pregnant women with type 1 diabetes and presented the results of 84 (intervention arm and control arm comprised 42 participants each) of them who successfully finished the trial in an academic hospital. The initiation of EPA and DHA supplementation or placebo started at randomization visit on gestational week 11–12. Blood samples were taken on the first (screening) visit to the clinic (1st trimester, between 8th and 10th gestational week, GW), then in the second trimester (19–24th GW) and third trimester (30th–33rd GW). On the delivery day, a blood sample was taken on fasting just before birth. The umbilical vein blood sample was taken shortly after the delivery.

**Results:**

We found a significant increase in the intervention group when compared the first and the third trimester for n-3 PUFAs concentration, 4.3 mg/L (3.3–7.6): 10.0 mg/L (7.1–13.7), *p* < .001. In the intervention group, the concentration of DHA in maternal vein serum was 11.4 mg/L (7.7–17.5), and in umbilical vein serum, it was 5.1 mg/L (3.0–7.7), which was significantly higher than that in the control group, maternal vein serum: median 9.2 mg/L(6.0–12.3), *p* = .03 and umbilical vein serum: median 3.4 mg/L (2.1–5.6), *p* = .009.

**Conclusion:**

The increased weight gain in pregnancy and concentration and proportions of DHA, n-3 PUFAs with a decreased proportion of AA, n-6 PUFAs, and AA/DHA ratio in maternal and umbilical vein serum summarize the effect of supplementation with EPA and DHA.

## 1. Introduction

Type 1 diabetes is an autoimmune disease because of selective beta-cell destruction and insulin secretion defects [1]. In addition to carbohydrate metabolism disturbances, we associate T1DM with maternal lipid content and composition [2]. A combination of n-3 PUFAs and pregnancy yields immunological tolerance and stimulates endogenous insulin production in women with T1DM [3]. Lower concentrations and lower proportions of n-3 PUFAs have been observed in neonates born to diabetic mothers [4,5]. Whalley et al. and Neuringer et al. have shown that intrauterine reduction in DHA concentration leads to learning difficulties afterward [6,7]. Docosahexaenoic acid plays a crucial role in developing nervous system cells and reduces preterm birth frequency [8]. The foetus depends on the transport of long-chain polyunsaturated fatty acids through the placenta. The placenta is dominant for the selective channelling of DHA from mother to foetus [9]. Maternal plasma-free fatty acids are an essential source of PUFAs for the foetus [2]. On the placental cell membrane, there are specific FABP proteins responsible for the transfer of PUFAs [9,10]. The transfer of these fatty acids is markedly determined and directed from the mother to the foetus. Several active transporters located on the surface of either the maternal or foetal side of the placenta regulate the transport of nutrients through the placenta [9]. The ability of fatty acids to pass through the placenta is vital for foetal brain development, foetal growth, and cardiovascular and pulmonary development. Proper transfer of fatty acids through the placenta in the foetus’s direction plays a significant role in healthy foetal development. It is a significant energy source in constructing the cell membrane and is an essential signalling precursor of cellular molecules. In term placentas, after the release of free fatty acids from VLDL or chylomicrons, fatty acids are transported and bound to FABP, which are further translocated to the cell [11–13].

N-3 PUFA supplementation in pregnancy has many benefits for both mother and baby. It reduces depression in pregnant women, prolongs pregnancy duration, reduces the risk of premature birth, and increases newborns’ weight [14,15]. An increase in IQ and cognitive function of 4-year-old children whose mothers took n-3 PUFA supplementation during pregnancy and lactation has been demonstrated [16].

This study aims to explore a) the influence of DHA and EPA supplementation on the change in the concentration and the percentage of individual FAs, total FAs, saturated fatty acids (SFAs), monounsaturated fatty acids (MUFAs), and n-6 polyunsaturated fatty acids (PUFAs) in maternal vein serum and umbilical vein serum. b) the impact of EPA and DHA supplementation on gestational weight gain (GWG), body mass index (BMI), and HbA1c during pregnancy. c) the impact of EPA and DHA supplementation on foetal growth. d) to calculate the ratio of fatty acids between maternal venous serum and umbilical vein serum as prioritizing transport of individual fatty acids from mother to foetus. e) a correlation of FAs in maternal and umbilical serum.

## 2. Subjects and methods

A prospective randomized, single-blinded, placebo-controlled trial (https://doi.org/10.1186/ISRCTN15203878) was undertaken at Referral Centre for Diabetes in Pregnancy Ministry of the Health Republic of Croatia, Department of Obstetrics and Gynaecology, Zagreb, University Hospital Centre, Zagreb, Croatia, from January 1, 2014, and September 30, 2018.

### 2.1. Ethics approval

The Ethics Committee at the Department of Obstetrics and Gynaecology (No. 021- 1/208 A-13) and the School of Medicine’s Ethics Committee, the University of Zagreb (No. 380-59-10106-15-168/87) approved the study. The trial was part of the scientific project approved by the Ministry of Science, Education and Technology of the Republic of Croatia, entitled Metabolic and Endocrine Changes in Pregnant Patients with Diabetes (No. 108-1080401-0386). All respondents were informed of the survey (“Notice to Respondent”) and signed the “Consent for Adult Participation in the Survey.”

### 2.2. Study design

**Figure 1.**
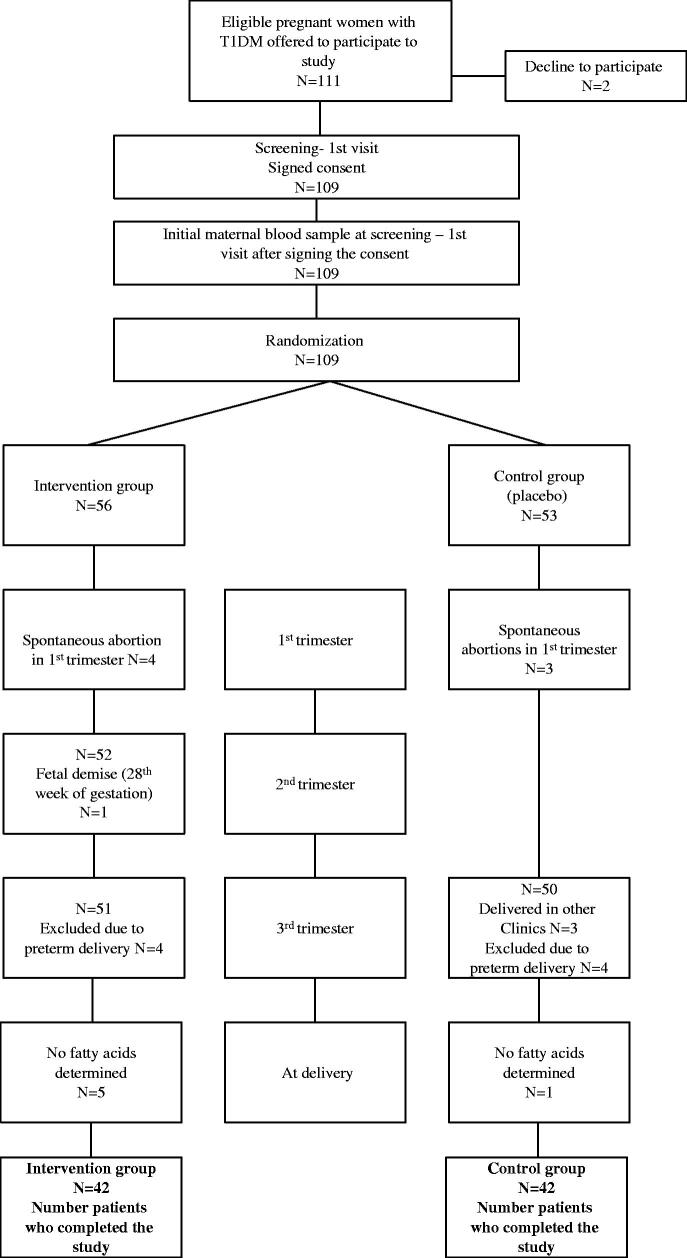
Flow chart of the participants.

One hundred and eleven eligible pregnant women with type 1 diabetes mellitus, according to the inclusion criteria between 8 and 10 gestational weeks, were informed and offered participation in the study (Figure 1). Two of them declined to be recruited. At this first (enrollment/screening) visit, 109 participants signed their informed consent, and the blood samples were collected. At the second randomization visit between gestational weeks 11 and 12, the computer-generated randomization program decided which pregnant woman with type-1 diabetes would be allocated to EPA and DHA (intervention arm) or placebo (control arm). Participants were randomly assigned in a 1:1 ratio.

Spontaneous abortion during the first trimester occurred in four women in the intervention group and three spontaneously aborted in the control group. During the second and third-trimester, dropouts in the intervention group were because of foetal demise and preterm delivery. In the control group, we lost seven subjects due to giving birth in other clinics or premature delivery. We did not collect the blood samples at delivery in 5 participants in the investigation arm and one in the control arm. Eighty-four pregnant women with T1DM were included in a prospective randomized, single-blinded, placebo-controlled trial. The number of participants was 42 subjects per arm who finished the trial, and their results are analyzed and presented in the study.

Forty-two of them were taking EPA and DHA twice a day (each capsule contains EPA 60 mg and DHA 308 mg), whereas the rest of 42 women were on the usual diabetic diet for pregnant women taking placebo twice a day (each capsule contains 2 mL corn oil; 948 mg linoleic acid and 18 mg α-linolenic acid). The starting of EPA and DHA supplementation into the pregnancy was between 11 and 12 weeks of pregnancy.

All participants received general advice on healthy eating before becoming pregnant (pre-pregnancy counselling for women with type 1 diabetes). Blood samples were analyzed from all pregnant women for fasting C-peptide (FC-peptide), fasting plasma glucose (FPG), HbA1c, concentration, and proportion of FAs in each trimester throughout pregnancy and after delivery. We analyzed umbilical vein blood for foetal C-peptide level, glucose concentration, insulin resistance, concentration, and proportion of FAs.

### 2.3. Inclusion criteria

The study included diabetic pregnant women who had no diabetes complications (proliferative retinopathy, nephropathy, chronic hypertension, and neuropathy), and the pregnancy progressed neatly and resulted in the delivery of healthy children/newborns.

Pregnant women were on intensified insulin therapy (a combination of basal-bolus analog insulins) and had well-controlled glycaemia.

### 2.4. Exclusion criteria

Women already taking prenatal supplements containing EPA and DHA were not eligible for the study. The exclusion criteria were spontaneous abortion, premature delivery, multiple pregnancies, genetic abnormalities, or foetal malformations.

### 2.5. Materials

The standards for gas chromatography (Supelco FAME MIX 37, PUFA No. 1, PUFA No. 3) are the product of the factories of Supelco Inc., Bellefonte, PA, USA. Organic solvents used for total lipid extraction were purchased from Sigma-Aldrich, Chemie GmbH, Steinheim, Germany, internal standard heptadecanoic acid, and anhydrous sodium sulphate 2,6-di-tert-butyl-4-methyl phenol (BHT) and concentrated hydrochloric acid.

Blood samples were taken on fasting in the morning from the left cubital vein into tubes with serum separation gel (Vacuette® tube 5 mL Z Serum Separator Clot Activator). All samples were centrifuged at 2000 g within one hour of sampling for 10 min. The serum was separated, dissolved in two tubes (cryotube), and stored in a freezer at 75 °C until further analysis. Blood samples were taken on the first screening visit to the clinic (1st trimester, between 9^th^ and 10^th^ week of pregnancy), then in the second trimester (19^th^–24^th^week) and third trimester (30^th^–33^rd^week). On the day of birth, a blood sample was taken on fasting just before birth. The umbilical vein blood sample was taken shortly after delivery before the placenta was removed.

Blood glucose, C-peptide concentrations, and glycated haemoglobin (HbA1c) content in maternal and umbilical vein blood were determined on the day of sampling, while total lipid analysis was subsequently performed.

Determination of C-peptide concentration (pmol/L) was performed at the Clinical Institute for Laboratory Diagnostics of Clinical Hospital Zagreb using the electrochemiluminescent method “ECLIA” on the Roche-Elecsys (Roche Diagnostics, Switzerland) autoanalyzer, reagent from the same manufacturer. Analytical sensitivity 0.003 nmol/L.

Glucose concentration (mmol/L) was determined by an enzymatic UV test (reference method with hexokinase) on a Roche Cobas C301 analyzer using reagents from the same manufacturer (Roche Diagnostics, Switzerland) at the Clinic for Women’s Diseases and Births. Analytical sensitivity 0.056 mmol/L.

Determination of HbA1c (%) by turbidimetric inhibition immunoassay on a Roche Cobas C501 analyzer of the same manufacturer (Roche Diagnostics, Switzerland) was performed Clinical Laboratory of Laboratory Diagnostics of Clinical Hospital Centre Zagreb.

Neonatal insulin resistance was calculated according to homeostasis Model Assessment 2 (HOMA2) [17] using software available online (http://homa.calculator.software.informer.com/2.2). The extraction of total lipids and the conversion of fatty acids to methyl esters was carried out at the Department of Chemistry and Biochemistry, School of Medicine, University of Zagreb. Total lipids were isolated from serum samples by the Folch extraction method with organic solvents [18].

The analysis of the composition of fatty acid methyl esters was performed by gas chromatography using an Agilent 7890B chromatograph (Agilent Technologies, CA, USA) equipped with a flame ionization detector (FID) at a laboratory service company, Sample Control Ltd. We used a capillary HP 88 column that had a length of 100 m, an internal diameter of 0.25 mm, and an active layer thickness of 0.20 µm (Agilent J&W, USA) to separate methyl esters. The instrument was equipped with an autosampler from the same manufacturer to apply a reduced sample volume (split/splitless system). The injector temperature was 200 °C, and the detector temperature was 250 °C.

The initial column temperature was 140 °C for the first 5 min, followed by an increase from 4 °C/min to 240 °C, maintained for 15 min. The sample (0.5 µL) was injected in split mode at a ratio of 1:100. With a purity of 6.0, helium was used as the carrier gas at a flow rate of 0.8 mL/min.

Identifying individual fatty acids was performed based on the retention time by comparing the retention time of known standards (PUFA No. 1, PUFA No. 3, C8-C20, Supelco 37 Component FAME mix, all Supelco, USA).

The fatty acid content was expressed as the relative weight (%) and mass concentration (mg/L). Quantitative determination of individual fatty acids was performed by comparing the peak area of the internal standard (heptadecanoic acid, C 17: 0, Sigma Aldrich, Germany) with the individual fatty acids’ peak area. Chromatogram processing was performed using an integrated computer program, Agilent 7890B GC (Agilent Technologies, CA, USA) in a trial with 80% power, a level of 0.05, and three tests of significance.

### 2.6. Sample size

For *post-hoc* power calculation, we tested the Bonferroni adjustment for multiple comparisons of n-3 PUFAs proportion in maternal vein serum. For a sample size of 42 participants in each study group and, *α* level of 0.05, a power (1-β) was 0.9683486. We also tested the *post-hoc* power difference mean of the percentage of n-3 PUFAs in both studied groups’ umbilical vein serum. For the sample size of 42 participants in each group and the *α* level of 0.05, a power (1-β) was 0.950473.

### 2.7. Statistical analysis

Statistical analyses were performed using the statistical package SPSS version 24 (IBM, Armonk, NY, USA). Absolute and relative frequencies represent categorical data. Numerical data are described by the mean (arithmetic mean) and standard deviation in the distributions following the normal, and in other cases by the mean (median) and the interquartile range’s limits. The Bonferroni adjustment test was used to examine the normality of the distribution of numerical variables. Student’s *t*-test tested group differences between normally distributed continuous variables, and differences between nonnormally distributed continuous variables were tested by the Mann–Whitney U test. Group differences between categorical variables were tested by the *χ*^2^ test and, if necessary, by Fisher’s exact test. The correlation between normally distributed numerical variables was evaluated by Pearson’s correlation coefficient (*r*). Data that were not normally distributed were log-transformed before analyses. Regression with the Spearman correlation coefficient (*r*_rho_) was performed for non-normal data. For repeated measurements of continuous data, the Wilcoxon signed-rank test was used. All *p* values ​​are two-sided. The significance level was set at *p* < .05.

## 3. Results

### 3.1. Maternal and neonatal characteristics

No significant difference between the two groups could be established concerning age, duration of diabetes, and the onset of type one diabetes. In the second and third trimesters of pregnancy, the intervention group participants gained significantly more weight compared to the controls. A significant decline in HbA1c was found in the control group over the trimesters, although the increase in BMI and weight gain between the first and third trimesters occurred in the same group. We could not establish any difference in the prevalence of neonatal macrosomia between the studied groups, as shown in Table 1. A significant decrease in HbA1c and an increase in maternal fasting C-peptide, pregnancy weight gain, weight gain difference, and BMI were found in the intervention group when comparing the first and third trimesters of pregnancy. No difference was found in pregnancy duration, neonatal weight and length, neonatal ponderal index, Apgar index at 1 and 5 min, umbilical artery pH, male/female newborn ratio, and macrosomia incidence, umbilical vein glucose and C-peptide concentration, and insulin resistance (IR HOMA-2) between studied groups.

**Table 1.** Maternal and neonatal characteristics.

	Intervention group (*n* = 42)	Control group (*n* = 42)	*p*	95% CI	
				Lower	Upper
Maternal characteristics					
Age (years)	29.4 ± 5.3	30.5 ± 5.2	.321	–1.134	3.420
Diabetes duration (years)	11.8 ± 6.9	13.8 ± 7.3	.176	–0.971	5.209
Age of onset type 1 DM	17.6 ± 7.5	16.6 ± 8.0	.565	–4.33821	2.38583
Body mass index in the first trimester (kg/m^2^)	22.9 ± 2.5^a^	23.5 ± 3.2^b^	.802	–1.7516	1.4334
Body mass index in the secondtrimester (kg/m^2^)	24.8 ± 2.9	24.6 ± 3.0	.726	–1.5566	1.0900
Body mass index in the third trimester (kg/m^2^)	27.1 ± 3.1^a^	26.3 ± 3.1^b^	.284	–2.1441	0.6366
Body mass index at the day of delivery (kg/m^2^)	27.7 ± 3.3	27.2 ± 3.2	.539	–1.8885	0.9957
Gestational weight gain in the first trimester (kg)	1.3 ± 2.1^c^	0.6 ± 2.8^d^	.226	–1.7413	0.1475
Gestational weight gain in the second trimester (kg)	6.5 ± 3.2	5.0 ± 3.4	**.045**	–2.8745	–0.0304
Gestational weight gain in the third trimester (kg)	12.8 ± 4.3^c^	9.9 ± 4.2^d^	**.002**	–4.7634	–1.0900
Gestational weight gain difference between the third and the first trimester (kg)	11.5 ± 4.0	9.2 ± 4.1	**.012**	–4.09539	–0.52604
Gestational weight gain at the day of delivery (kg)	14.3 ± 4.8	12.4 ± 4.2	.067	–3.7815	0.1291
HbA_1C_ in the first trimester (%)	6.8 ± 1.0^f^	6.6 ± 1.2^g^	.415	–0.6948	0.2898
HbA_1c_ in the second trimester (%)	5.7 ± 0.6	5.6 ± 0.5	.367	–0.3648	0.1362
HbA_1c_ in the third trimester (%)	6.0 ± 0.8^f^	5.8 ± 0.7^g^	.229	–0.5376	0.1308
HbA_1c_ difference between the third and the first trimester (%)	–0.87 ± 0.9	–0.85 ± 1.1	.954	–0.4325	0.4581
Smoking no/yes *n* (%)	35/7 (83.3/16.7)	37/5 (88.1/11.9)	.378	0.744	0.766
Primipara/multipara *n* (%)	24/18 (57.1/42.9)	27/15 (64.3/35.7)	.503	0.643	0.668
Maternal serum measurements					
FC-peptide concentration in the first trimester (pmol/L)	10.0^h^ (10.0–60.0)	10.0 (10.0–30.0)	.925		
FC-peptide concentration in the second trimester (pmol/L)	10.0 (10.0–110.0)	10.0 (10.0–20.0)	.131		
FC-peptide concentration in the third trimester (pmol/L)	20.0^h^ (10.0–150.0)	10.0 (10.0–30.0)	**.045**		
FC-peptide concentration after delivery (pmol/L)	20.0 (10.0–100.0)	10.0 (10.0–20.0)	.210		
FP glucose in the first trimester (mmol/L)	5.2 ± 1.9	5.1 ± 1.9	.854	–0.9194	0.7633
FP glucose in the second trimester (mmol/L)	4.1 ± 1.3	5.0 ± 2.0	**.014**	0.1893	1.6488
FP glucose in the third trimester (mmol/L)	5.1 ± 2.0	4.7 ± 1.2	.293	–1.0964	0.3345
FP glucose at the time of delivery (mmol/L)	5.4 ± 1.7	5.4 ± 2.0	.926	–0.7714	0.8476
Neonatal characteristics					
Gestational age	38.6 ± 0.8	38.4 ± 0.8	.285	–0543	0.162
Birth weight (g)	3488.8 ± 496.8	3517.5 ± 455.5	.781	–176.147	233.528
Birth length (cm)	49.2 ± 2.0	49.5 ± 2.0	.487	–0.529	1.101
Ponderal index (g) × 100/ (cm)^3^	2.9 ± 0.3	2.9 ± 0.2	.604	–0.15144	0.08849
Apgar score at 1 min	9.8 ± 0.3	9.9 ± 0.4	.394	–0.221	0.411
Apgar score at 5 min	9.9 ± 0.5	9.9 ± 0.2	.563	–0.115	0.210
pH umbilical artery blood	7.24 ± 0.07	7.24 ± 0.07	>.9	–0.03518	0.03500
Sex ratio: male: female n (%)	25/17 (59.5/40.5)	21/21 (50.0/50.0)	.511	0.499	0.524
Macrosomic: eutrophic neonates	5/37 (11.9/88.1)	7/35 (16.7/83.3)	.756	0.749	0.771
Umbilical vein blood measurements					
C-peptide (pmol/L)	690.0 (510.0–980.0)	800.0 (430.0–1270.0)	.328		
Glucose (mmol/L)	4.6 ± 2.2	4.9 ± 1.6	.451	–0.5283	1.1771
IR HOMA-2	1.5 (1.1–2.2)	1.9 (0.9–2.9)	.242		

FP glucose: fasting plasma glucose; FC-peptide: fasting C-peptide; T1DM: type 1 diabetes mellitus; BMI: body mass index; data are mean (SD) unless otherwise indicated, data are median (1^st^ and 3^rd^ quartiles), *t*-test was used to test the statistical difference between two groups, or Mann–Whitney U test, *p* values in bold are significant, 1^st^ and 3^rd^ trimester of pregnancy. ^a,b,c,d,h^*p*< .001; *p* Statistically significant at the Bonferroni-corrected α. ^f,g^*p*< .05 Wilcoxon signed-rank test; *p* Statistically not significant at the Bonferroni-corrected α.

### 3.2. Concentration of fatty acids in the two study groups

A significantly higher concentration of α-linolenic acid (C18: 3n-3; *p* = .025) was found in the intervention group in the first and second trimester than in the control group. A higher DHA and n-3 PUFA concentrations were found in the second and third trimesters of pregnancy in the intervention group (*p* = .004 and *p* = .001) than in the control group. The concentration of γ-linolenic acid (C18: 3 n-6) was higher in the first and second trimester in the intervention group (*p* = .025 and *p* = .001) compared to the control group.

The concentrations of FAs between the first and third trimester of pregnancy were compared in the intervention group. A significant increase was confirmed for total fatty acids, palmitic acid (C16: 0), stearic acid (C18:0), SFAs, palmitoleic acid (C16: 1 n-7), oleic acid (C18:1 n-9), MUFAs, EPA (C20:5 n-3), DHA (C22: 6n-3) and n-3 PUFAs. Accordingly, we compared the concentrations of FA between the first and third trimester in the control group. A significant increase was measured for total fatty acids, stearic acids (C18:0), SFA, palmitoleic acid (C16:1 n-7), oleic acid (C18: 1n-9), MUFAs, EPA (C20:5 n-3), DHA (C22:6 n-3), n-3 PUFAs, linoleic acid (C18:2 n-6), arachidonic acid (C20:4 n-6), and n-6 PUFAs (Table 2).

**Table 2.** The concentration of fatty acids in two study groups of pregnant women with type 1 diabetes.

	Intervention group (*n* = 42)	Control group (*n* = 42)	*p*
Fatty acids (mg/L)			
Total fatty acids in the first trimester of pregnancy	276.9^a^(214.8–350.2)	227.9^b^(157.7–328.8	.115
Total fatty acids in the second trimester of pregnancy	394.4 (317.8–486.7)	380.8 (248.5–428.1)	.233
Total fatty acids in the third trimester of pregnancy	506.5^a^ (357.9–422.6)	422.6^b^ (289.1–596.1)	.112
Palmitic acid (C16:0) in the first trimester	69.3^c^(54.2–86.1)	59.6 (45.2–85.2)	.188
Palmitic acid (C16:0) in the second trimester	109.1 (76.8–129.1)	104.3 (67.1–117.0)	.266
Palmitic acid (C16:0) in the third trimester	142.4^c^(98.2–177.1)	121.9 (87.8–165.8)	.168
Stearic acid (C18:0) in the first trimester	24.9^d^(17.6–31.8)	19.9^e^(12.9–31.5)	.089
Stearic acid (C18:0) in the second trimester	30.4 (24.2–40.0)	29.7 (17.5–33.0)	.096
Stearic acid (C18:0) in the third trimester	34.9^d^ (24.3–48.6)	28.8^e^ (20.1–41.1)	.137
SFAs in the first trimester	101.2^f^ (77.4–130.0)	79.2^g^ (59.6–126.3)	.125
SFAs in the second trimester	150.6 (108.0–188.0)	144.5 (93.9–176.1)	.330
SFAs in the third trimester	196.6^f^ (133.4–237.5)	165.8^g^ (117.2–226.1)	.233
Palmitoleic acid (C16:1 n-7) in the first trimester	2.9^h^ (2.0–4.6)	2.9^i^ (1.5–3.6)	.316
Palmitoleic acid (C16:1 n-7) in the second trimester	5.0 (3.5–6.4)	3.6 (2.2–5.9)	.145
Palmitoleic acid (C16:1 n-7) in the third trimester	6.6^h^ (4.4–9.2)	5.5^i^ (3.5–8.2)	.050
Oleic acid (C18:1 n-9) in the first trimester	45.8^j^ (32.6–64.3)	37.4^k^ (20.6–53.5)	.082
Oleic acid(C18:1 n-9) in the second trimester	63.2 (43.2–83.1)	60.0 (40.0–72.3)	.083
Oleic acid(C18:1 n-9) in the third trimester	89.1^j^ (61.2–121.3)	74.1^k^ (43.1–107.1)	.091
MUFAs in the first trimester	51.7^l^ (38.4–72.3)	42.2^m^ (23.1–58.4)	.061
MUFAs in the second trimester	71.5 (51.7–99.8)	65.6 (38.6–78.7)	.059
MUFAs in the third trimester	101.5^l^ (70.9–137.0)	85.1^m^ (50.5–114.2)	.133
α-linolenic acid (C18:3 n-3) in the first trimester	0.0 (0.0–2.5)	0.0 (0.0–2.0)	**.025**
α-linolenic acid (C18:3 n-3) in the second trimester	2.1 (0.8–2.3)	1.8 (0.0–2.5)	**<.001**
α-linolenic acid (C18:3 n-3) in the third trimester	1.3 (0.0–1.9)	1.0 (0.0–1.6)	.089
Eicosapentanoic acid in the first trimester (C20:5 n-3)	2.3^n^ (1.6–3.2)	2.2^o^ (0.8–3.5)	.622
Eicosapentanoic acid in the second trimester (C20:5 n-3)	2.9 (2.1–4.4)	4.0 (2.0–4.4)	.639
Eicosapentanoic acid in the third trimester (C20:5 n-3)	3.9^n^ (2.5–5.2)	3.4^0^ (2.3–5.4)	.525
Docosahexaenoic acid in the first trimester (C22:6 n-3)	4.3^p^ (3.3–7.0)	3.7r^t^ (2.5–5.8)	.157
Docosahexaenoic acid in the second trimester (C22:6 n-3)	10.0 (7.1–13.7)	7.4 (5.1–9.2)	**.004**
Docosahexaenoic acid in the third trimester (C22:6 n-3)	13.4^p^ (7.2–18.1)	7.4^r^ (5.6–11.4)	**.001**
n-3 PUFA in the first trimester	8.6^s^ (5.8–12.2)	7.0^t^ (4.1–13.0)	.257
n-3 PUFA in the second trimester	18.8 (13.7–20.6)	13.0 (9.6–16.4)	**.003**
n-3 PUFA in the third trimester	20.8^s^ (14.0–28.9)	13.4^t^ (8.7–19.8)	**.002**
Linoleic acid in the first trimester(C18:2 n-6)	75.9^u^ (62.5–110.3)	72.0^v^ (48.8–103.0	.212
Linoleic acid in the second trimester (C18:2 n-6)	123.2 (83.0–143.4)	110.4 (75.4.138.2)	.423
Linoleic acid in the third trimester(C18:2 n-6)	147.1^u^ (110.2–177.9)	127.5^v^ (81.8–187.5)	.317
γ-linolenic acid (in the first trimester C18:3 n-6)	1.3 (0.0–1.6)	0.0 (0.0–1.2)	**.025**
γ-linoleic acid (in the second trimester C18:3 n-6)	1.3 (1.0–1.7)	0.0 (0.0–1.2)	**.001**
γ-linolenic acid (in the third trimester C18:3 n-6)	1.3 (0.0–2.0)	1.0 (0.0–1.6)	.089
Arachidonic acid in the first trimester (C20:4 n-6)	21.5^w^ (13.6–28.6)	20.1^x^ (13.5–30.3)	.774
Arachidonic acid in the second trimester (C20:4 n-6)	28.6 (20.9–36.29	26.8 (18.6–33.7)	.808
Arachidonic acid in the third trimester (C20:4 n-6)	29.3^w^ (22.6–43.0)	31.8^x^ (20.7–37.0)	.680
n-6 PUFA in the first trimester	105.1^y^ (83.3–147.6)	99.6^z^ (68.1–136.3)	.266
n-6 PUFA in the first second	159.7 (117.9–194.8)	146.9 (97.0–184.1)	.444
n-6 PUFA in the third trimester	188.8^y^ (139.7–243.6)	167.2^z^ (113.8–246.2)	.337

*p* values in bold are significant, 1^st^ and 3^rd^ trimester of pregnancy ^a,b,c,d,e,f,g,h,i,j,k,l,m,n,o,p,r,s,t,u,w,z^*p*< .001; Wilcoxon signed-rank test, *p*Statistically significant at the Bonferroni-corrected α.

### 3.3. The proportion of fatty acids in the two study groups

A higher proportion of DHA, n-3 PUFAs, and γ-linolenic acid (C18: 3 n-6) was found in the second and third trimesters of pregnancy. A lower proportion of linoleic acid (C18: 2 n-6), n-6 PUFAs, and AA/DHA was measured in the intervention group than in the control group. The proportions of FAs between the first and third trimesters of pregnancy were compared in the intervention group. A significant increase was found for palmitic acid (C16: 0), DHA (C22: 6n-3), n-3 PUFAs, and a significant reduction was found for stearic acid (C18: 0), arachidonic acid (C20: 4n-6), n-6 PUFAs, and AA/DHA ratio. Accordingly, we compared the proportions of FA between the first and third trimesters in the control group. A significant decrease was found for arachidonic acid (C22: 4n-6) (Table 3).

**Table 3.** The proportion of fatty acids in two study groups of pregnant women with type 1 diabetes pregnant women.

Ta	Intervention group (*n* = 42)	Control group (*n* = 42)	*p*
Palmitic acid (C16:0) in the first trimester	25.4^a^ (24.4–26.2)	26.1 (24.2–27.0)	.131
Palmitic acid (C16:0) in the second trimester	26.9 (26.0–28.5)	27.0 (25.8–28.8)	.799
Palmitic acid (C16:0) in the third trimester	28.2^a^ (27.1–29.2)	27.7 (26.5–29.2)	.168
Stearic acid (C18:0) in the first trimester)	8.8^b^ (8.4–9.5)	8.9 (8.0–9.2)	.287
Stearic acid (C18:0) in the second trimester	8.0 (7.6–8.7)	7.8 (7.3–8.5)	.299
Stearic acid (C18:0) in the third trimester	7.3^b^ (6.6–7.7)	7.1 (6.5–7.8)	.970
SFAs in the first trimester	36.2 (35.5–37.5)	37.1 (35.6–38.5)	.117
SFAs in the second trimester	37.4 (36.4–38.6)	37.4 (35.7–39.7)	.938
SFAs in the third trimester	37.6 (36.8–38.9)	37.4 (36.5–39.0)	.893
Palmitoleic acid (C16:1 n-7) in the forst trimester	1.1 (1.0–1.4)	1.1 (0.7–1.5)	.863
Palmitoleic acid (C16:1 n-7) in the second trimester	1.2 (0.9–1.7)	1.1 (0.8–1.4	.289
Palmitoleic acid (C16:1 n-7) in the third trimester	1.4 (1.0–2.0)	1.3 (0.5–1.5)	.204
Oleic acid (C18:1 n-9) in the first trimester	16.2 (15.2–19.1)	15.7 (14.2–18.0)	.242
Oleic acid(C18:1 n-9) in the second trimester	16.4 (15.0–17.8)	15.9 (14.6–17.7)	.226
Oleic acid(C18:1 n-9) in the third trimester	17.8 (16.2–19–3)	17.2 (16.6–19.2)	.996
MUFA in the first trimester	18.4 (17.2–21.6)	17.8 (16.0–20.4)	.117
MUFAs in the second trimester	19.0 (17.3–20.6)	17.8 (16.3–20.1)	.089
MUFAs in the third trimester	20.2 (18.5–21.7)	20.0 (18.1–21.6)	.507
α-linolenic acid (C18:3 n-3) in the first trimester	0.0 (0.0–0.6)	0.0 (0.0–0.7)	.805
α-linolenic acid (C18:3 n-3) in the second trimester	0.5 (0.3–0.6)	0.5 (0.0–0.5)	.255
α-linolenic acid (C18:3 n-3) in the third trimester	0.5 (0.4–0.7)	0.5 (0.4–0.6)	.152
Eicosapentanoic acid in the first trimester (C20:5 n-3)	0.9 (0.7–1.0)	0.9 (0.5–1.1)	.760
Eicosapentanoic acid in the second trimester (C20:5 n-3)	0.8 (0.6–0.9)	1.0 (0.7–1.1)	**.017**
Eicosapentanoic acid in the third trimester (C20:5 n-3)	0.7 (0.6–1.9)	0.9 (0.6–1.1)	.290
Docosahexaenoic acid in the first trimester (C22:6 n-3)	1.7^c^ (1.3–2.1)	1.6 (1.4–2.0)	.672
Docosahexaenoic acid in the second trimester (C22:6 n-3)	2.7 (2.2–3.4)	2.0 (1.8–2.4)	**.004**
Docosahexaenoic acid in the third trimester (C22:6 n-3)	2.7^c^ (2.2–3.3)	1.9 (1.4–2.3)	**<.001**
n-3 PUFA in the first trimester	3.1^d^ (2.5–4.1)	3.1 (2.3–4.1)	.763
n-3 PUFA in the second trimester	4.3 (3.5–5.2)	3.4 (2.9–3.8)	**.001**
n-3 PUFA in the third trimester	4.3^d^ (3.3–5.0)	3.1 (2.7–3.9)	**<.001**
Linoleic acid in the first trimester(C18:2 n-6)	29.4 (27.0–31.6)	29.6 (28.0–32.6	.355
Linoleic acid in the second trimester (C18:2 n-6)	27.9 (25.9–30.9)	30.8 (27.5.32.3)	**.037**
Linoleic acid in the third trimester(C18:2 n-6)	27.9 (25.7–30.1)	30.2 (28.0–31.8)	**.013**
γ-linolenic acid in the first trimester (C18:3 n-6)	0.3 (0.0–0.6)	0.0 (0.0–0.3)	**.037**
γ-linolenic acid in the second trimester	0.4 (0.2–0.5)	0.0 (0.0–0.3)	**.001**
γ-linolenic acid in the third trimester (C18:3 n-6	0.2 (0.0–0.4)	0.2 (0.0–0.3)	.391
Arachidonic acid in the first trimester (C20:4 n-6)	8.2^e^ (6.6–9.7)	8.4^f^ (7.6.5–9.3)	.301
Arachidonic acid in the second trimester (C20:4 n-6)	7.6 (6.3–8.2)	7.8 (6.7–8.8)	.096
Arachidonic acid in the third trimester (C20:4 n-6)	6.5^e^ (5.7–7.2)	7.0^f^ (6.0–8.1)	.060
n-6 PUFA in the first trimester	41.1^g^ (37.9–42.8)	41.5 (39.9–43.3)	.204
n-6 PUFA in the second trimester	38.2 (36.5–40.0)	41.3 (37.6–43.1)	**.013**
n-6 PUFA in the third trimester	36.9^g^ (35.3–39.0)	39.7 (38.2–40.9)	**.002**
AA/DHA in the first trimester	4.8^h^ (3.3–6.0)	5.1 (4.1–6.7)	.214
AA/DHA in the second trimester	2.8 (2.2–3.4)	3.8 (3.3–4.6)	**<.001**
AA/DHA in the third trimester	2.3^h^ (2.0–3.0)	3.8 (3.0–4.5)	**<.001**
LA/AA in the first trimester	3.5^i^ (2.9–5.0)	3.8^j^ (3.2–4.0)	.764
LA/AA in the second trimester	4.0 (3.3–4.8)	4.0 (3.3–4.5)	.731
LA/AA in the third trimester	4.3^i^ (3.9–5.2)	4.5^j^ (3.6–5.3)	.792

LA: linoleic acid (C18:2n-6); AA: arachidonic acid (C:20:4n-6); *p* values in bold are significant, 1^st^ and 3^rd^ trimester of pregnancy ^a,b,c,d,e,f,g,h,i,j^*p*<.001;Wilcoxon signed-rank test, *p* Statistically significant at the Bonferroni-corrected α.

### 3.4. Concentration difference of fatty acids between the third and the first trimester of pregnancy in two studied groups

Supplemental Tables 1 and 2 show the difference in concentration and proportion of fatty acids between the first and third trimester. The intervention group had a significantly more large difference for DHA and n-3 PUFA than the control group.

### 3.5. Concentration and proportion of fatty acids in maternal and umbilical vein serum

Table 4 shows the FA concentrations in maternal serum, umbilical vein, and FA concentration ratios between maternal blood and umbilical vein. Significantly higher concentrations were found in the intervention group for palmitoleic acid (C16: 1 n-7) and DHA (C22: 6 n-3) compared to the control group. Significantly higher values ​​were found in the umbilical vein serum of the stearic acid, DHA, and n-3 PUFAsin the intervention group (C18: 0) *p* = .030, DHA (C22: 6n-3) *p* = .009 and n-3 PUFAs, *p* = .001 compared to the control group. The FA concentration ratios between the maternal and umbilical veins ranged from 1.0 for γ-linolenic acid (C18: 3n-6) to 8.0 for linoleic acid (C18: 2n-6). The concentration ratio between maternal and umbilical vein serum for DHA was 2.1 (1.5–2.8) for the intervention group and 2.4 (1.7––3.0) for the control group and AA was 1.4 (1.1–2.2) for the intervention group and 1.4 (1.1–2.0) for the control group. A higher concentration ratio between maternal blood and umbilical vein (median 7.5 and 8.0) was obtained for linoleic acid (C18: 2n-6). The proportion of palmitoleic acid (C16:1n-7), α-linoleic acid (C18:3n-3), DHA (C22:6n-3), and n-3 PUFAs were significantly higher in the serum of mothers from the intervention group compared to the control group directly before childbirth. In the umbilical vein serum, the proportions of SFAs and AA were higher in the control group than in the intervention group. In contrast, the proportions of γ-linolenic acid (C18: 3 n-6), DHA, and n-3 PUFA were higher in the intervention group, as shown in Table 3. A significantly lower proportion ratio between maternal and umbilical vein serum was found in the intervention group compared to the control for n-3 PUFAs [0.85 (0.68–0.98): 0.97 (0.8–1.24), *p* = .044], as shown in Supplemental Table 3.

**Table 4.** The concentration of fatty acids in maternal and umbilical vein serum at the time of birth.

	Maternal serum at birth			Umbilical vein serum			Ratio maternal serum: umbilical vein serum		
Fatty acids (mg/L)	Intervention group (*n* = 42)	Control group (*n* = 42)	*p*	Intervention group (*n* = 42)	Control group (*n* = 42)	*p*	Intervention group (*n* = 42)	Control group (*n* = 42)	*p*
Total fatty acids	518.7 (355.4–489.6)	463.1 (366.1–570.1)	.543	184.2 (174.1–243.3)	138.7 (106.3–189.2)	.189	3.1 (2.3–4.6)	3.5 (3.3–4.9)	.741
Palmitic acid (C16:0)	150.3 (99.4–195.4)	140.2 (103.3–192.9)	.610	44.5 (33.9–73.8)	42.0 (30.0–57.6)	.190	3.1 (2.1–4.5)	3.2 (2.2–4.7)	.631
Stearic acid (C18:0)	35.8 (25.7–47.5)	35.6 (25.3–46.7)	.089	18.5 (12.3–28.0)	13.8 (9.1–20.7)	**.030**	2.0 (1.4–2.3)	2.1 (1.4–2.5)	.551
SFAs (C16:0 + C18:0)	201.8 (113.2–261.8	159.8 (131.9–263.0)	.747	71.9 (50.3–106.8)	62.5 (42.3–89.9)	.215	2.8 (2.0–4.0)	2.9 (1.9–4.24)	.592
Palmitoleic acid (C16:1 n-7)	9.2 (6.9–13.9)	6.5 (4.2–10.7)	**.012**	6.0 (3.9–7.7)	5.0 (3.0–6.2)	.060	1.6 (1.2–2.1)	1.5 (0.7–3.2)	.610
Oleic acid (C18:1 n-9)	99.0 (60.0–135.7)	91.6 (67.6–121.1)	.623	23.0 (17.5–34.0)	21.1 (9.0–29.7)	.238	4.2 (3.0–5.9)	4.4 (2.8–10.2	.719
MUFAs	111.4 (70.3–160.4)	103.4 (74.9–137.7)	.463	31.7 (23.7–45.7)	26.3 (14.0–39.0)	.142	3.6 (2.6–5.0)	3.9 (2.3–6.0)	.719
α-linolenic acid (C18:3 n-3)	2.9 (1.9–4.0)	2.4 (0.9–3.3)	.117	0.0 (0.0–0.0)	0.0 (0.0–0.0)	NA			
Eicosapentanoic acid (C20:5 n-3)	3.2 (2.3–4.6)	3,6 (2.2–5,8)	.546	1.8 (0.0–2.4)	0.0 (0.0–2.1)	.066	1.6 (1.1–2.1)	2.0 (1.1–3.1)	.290
Docosahexaenoic acid (C22:6 n-3)	11.4 (7.7–17.5)	9.2 (6.0–12.3)	**.030**	5.1 (3.0–7.7)	3.4 (2.1–5.6)	**.009**	2.1 (1.5–2.8)	2.4 (1.7–3.0)	.589
n-3 PUFA	11.3 (7.7–17.5)	9.2 (6.0–12.3)	.062	5.1 (3.0–7.7)	3.4 (2.1–5.6)	**.001**	2.1 (1.5–2.8)	2.4 (1.7–3.9)	.214
Linoleic acid (C18:2 n-6)	135.7 (97.5–193.8)	128.6 (89.3–187.3)	.648	17.8 (10.9–25.3)	15.5 (18.6–22.6)	.213	7.5 (4.9–11.2)	8.0 (5.5–12.6)	.728
γ-linolenic acid (C18:3 n-6)	1.2 (0.0–1.4)	0.0 (0.0–1.6)	.182	1.0 (0.0–1.4)	0.0 (0.0–0.3)	**<.001**	1.0 (0.7–1.4)	0.9 (0.0–2.0)	.887
Arachidonic acid (C20:4 n-6)	29.4 (22.2–40.2)	30.8 (20.0–43.3)	.915	19.3 (12.1–30.3)	20.4 (13.1–27.3)	.889	1.4 (1.1.2.2)	1.4 (1.1–2.0)	.737
n-6 PUFA	184.2 (123.3–247.0)	176.9 (119.2–240.8)	.655	43.5 (32.2–67.7)	43.0 (32.1–58.9)	.368	3.7 (3.1–5.4)	3.7 (2.6–5.5)	.944

*p* values in bold are significant.

### 3.6. Impact of HbA1c levels, GWG and n-6 PUFAs on newborn’s macrosomia

Mothers who gave birth to macrosomic newborns had significantly higher HbA1c levels in all three trimesters of pregnancy, a higher overall weight gain, and their newborns had a lower umbilical vein serum n-6 PUFAs concentration, all shown in Supplemental Table 4.

### 3.7. Nonparametric correlation between the maternal vein and umbilical vein serum of DHA and AA

Supplemental Figures 1 and 2 show a high significant nonparametric correlation coefficient of arachidonic acid (AA) (*r*_rho_= 0.420: *p* < .001) and docosahexaenoic DHA *(r*_rho_=0.550: *p* < .001)concentration between maternal and umbilical venous serum.

## 4. Discussion

### 4.1. Maternal and foetal effects of EPA and DHA supplementation

The gestational weight gain in the second and third trimesters was significantly higher in the intervention group than in the control group. Some studies have found a negative association between n-3 PUFA concentrations and body weight or adipose tissue volume [19,20]; other studies [21–23] have found an association between n-3 PUFA concentrations and abdominal obesity which is in line with our study. Studies in experimental animals have shown that dietary supplementation with ALA, EPA, and DHA reduces obesity [24]. A diet rich in n-6 PUFAs increases AA and its endocannabinoid metabolites, enhancing food consumption and obesity development [25–27]. The adipogenic effect of n-6 PUFAs can be prevented by consuming sufficient amounts of EPA and DHA to reduce AA in cellular phospholipids and endocannabinoid synthesis [28], which we did not find in our study.

Our study results show no effect of the total fatty acid concentration on the child's birth weight. A smaller proportion of n-6 PUFAs was found in the umbilical vein serum of macrosomic neonates than eutrophic neonates. Makrides et al. [29], in a randomized study with n-3 PUFA supplementation in healthy pregnant women, found no difference in birth weight or prevalence of neonatal macrosomia compared to the controls, which is in line with our study. According to Pedersen's hypothesis, foetuses from mothers with T1DM exhibit accelerated growth because increased maternal glucose transmission from the placenta to the foetus causes foetal hyperinsulinemia [30,31]. With strict metabolic control, we reduced the incidence of neonatal macrosomia to only 14%. In contrast to our results in reducing neonatal macrosomia, Evers et al. [32] found a high prevalence (45.1%) of neonatal macrosomia despite, in their opinion, optimal metabolic control. Mothers of macrosomic infants had elevated HbA1c levels in all three trimesters of pregnancy and higher total weight gain than mothers of eutrophic infants.

In our recent studies, macrosomic foetuses of mothers with type 1 diabetes had higher insulin, IR, and leptin levels. They manifested substantial differences in the total FA concentrations in the umbilical vein and artery, demonstrating an increased deposition of fatty tissue than those from the control group. Macrosomic foetuses from healthy pregnant women had normal blood glucose levels and non-elevated insulin and leptin levels [33,34].

### 4.2 C-Peptide concentration in pregnant women with type 1 diabetes mellitus

Comparing fasting C-peptide concentrations across all three trimesters, we found a significant increase from the first to the third trimester in the intervention group. This finding is consistent with our previous research showing that EPA and DHA supplementation in pregnancy with type 1 diabetes increases the C-peptide concentration [3] and is consistent with the reported increase in C-peptide throughout pregnancy Nielsen et al. [35].

### 4.3. Change in FA concentration and proportion during pregnancy

During pregnancy, the concentrations of total FAs, SFAs, MUFAs, n-6, and n-3 PUFAs and individual FAs in both studied groups increased significantly, which is in agreement with Al MD’s et al. study [36]. Pregnant women in the intervention group had a significantly higher increase in the concentration of DHA and n-3 PUFAs in the second and third trimester of pregnancy compared to the control group, which was expected given the EPA’s compensation DHA in the diet. In the intervention group, the proportions of palmitic and stearic acid, SFAs, DHA, and n-3 PUFAs increased during pregnancy, and the proportions of AA, n-6 PUFAs, and AA/DHA decreased significantly. The decrease in the proportion of AA, n-6 PUFAs, and the AA/DHA ratio increased DHA and n-6 ​​PUFAs in the control group. This study shows that supplementation with 120 mg/day EPA and 616 mg/day DHA throughout pregnancy significantly alters the fatty acid concentration and proportion of maternal and umbilical vein serum. Arachidonic acid derivatives have an inflammatory effect, increase platelet aggregation, induce smooth muscle cell contraction, and are associated with many diseases. The increased proportion of n-3 PUFAs in cell membrane phospholipids reduces the availability of arachidonic acid and inflammatory metabolite production. EPA is a precursor for the synthesis of prostaglandins, thromboxanes, prostacyclin series three, and leukotrienes series five, which have anti-inflammatory activity. Additionally, EPA and DHA synthesize resolvins and protectins that have potent anti-inflammatory activity. In the intervention group, pregnant women had higher concentrations and a higher proportion of DHA and total n-3 PUFAs, as expected, given EPA and DHA supplementation.

The total amount of FA did not differ in umbilical vein serum between the study groups. Higher concentrations of stearic acid (C18: 0), γ-linolenic acid (C18: 3 n-6), DHA, and n-3 PUFAs were found in the intervention group compared to the control. We consider that the increased concentration of stearic acid (C18: 0) in the serum of the intervention group’s umbilical vein results from increased foetal synthesis. Significantly higher concentrations and proportions of DHA and n-3 PUFAs and a reduced AA proportion and AA/DHA ratio in umbilical vein serum were found in the intervention group than in the control group. Comparing the ratios of individual FA concentrations between maternal and umbilical veins, we found no difference between the study groups. The lowest ratios were found for γ-linolenic acid (C18: 3 n-6), AA, EPA, and DHA, which means that these fatty acids pass in higher amounts than other FAs. The highest ratio was found for linoleic acid (C18: 2 n-6) (7.5 and 8.0), which means that this fatty acid passes in smaller amounts from the mother across the placenta to the foetus (biomagnification increased AA transport and less for LA).

The placenta is essential for selective AA and DHA canalisation from the mother to the foetus; the evidence is a high coefficient of correlation between AA and DHA in the mother and the foetus (*r*_rho_=0.550; *r*_rho_=0.420, all *p* < .001, Supplemental Figures 1 and 2).

There is no consensus recommendation for n-3 LC-PUFA supplementation in pregnant and lactating women. Some authors recommend a daily DHA intake of 300 mg during pregnancy and lactation, whereas others suggest 200–300 mg DHA daily [7,37]. European Food Safety Authority (EFSA) allows reaching the Dietary Reference Value for LC-PUFA (250 mg EPA plus DHA, plus an, additional 100 − 200 mg of DHA) either by food and supplements. Helland IB et al. prescribed the pregnant women supplementation of DHA 1183 mg/day and EPA 803 mg/day [16]. This study indicated that maternal supplementation with EPA and DHA during pregnancy and lactation improved children’s intelligence at four years [16].

The participants included in our study group received 616 mg DHA daily. It significantly increased the concentration and proportion of DHA, n-3 PUFAs in total lipids, decreased proportion of AA and n-6 PUFAs, and the decreased ratio of AA/DHA in maternal and foetal serum.

#### 4.3.1. Strengths and limitations of this study

Our study is the first one investigating the effects of EPA and DHA supplementation during pregnancy in women with type 1 diabetes to the best of our knowledge. The concentration of individual fatty acids assessed from the first trimester of pregnancy to delivery was the study’s strength. We have compared the fatty acid concentrations with GWG, HbA1c, C-peptide in the umbilical vein and explored their influence on foetal growth. We did not use a food frequency questionnaire (FFQ) to get the FAs intake information in our participants, which we consider a limitation of the study. On the other hand, diabetic patients in Croatia are controlled for almost 50 years in our State Referral Centre for Diabetes in Pregnancy and well known as patients of high compliance when participating in clinical trials. That reassured us that the vast majority of participants would previously ask for consultations if they decided to deviate from the study protocol. In conclusion, the increased concentrations and proportions of DHA and n-3 PUFAs with a decreased proportion of AA, n-6 PUFAs, and AA/DHA ratio measured in the maternal vein and umbilical vein serum summarize the effect of supplementation with EPA and DHA. According to this trial results, diabetic mothers and their newborns may benefit from EPA and DHA supplementation.

## Supplementary Material

Supplemental Material

## References

[1] Expert Committee on the Diagnosis and Classification of Diabetes Mellitus. Report of the Expert Committee on the Diagnosis and Classification of Diabetes Mellitus. Diabetes Care. 1997;20:1183–1197. 9203460 10.2337/diacare.20.7.1183

[2] Herrera E. Metabolic changes in diabetic pregnancy. In Djelmis J, Desoye G, Ivanisevic M, editors. Diabetology of pregnancy. Basel: Karger; 2005. p. 34–45.

[3] Horvaticek M, Djelmis J, Ivanisevic M, et al. Effect of eicosapentaenoic acid and docosahexaenoic acid supplementation on C-peptidepreservation in pregnant women with type-1 diabetes: a randomized placebo-controlled clinical trial. Eur J Clin Nutr. 2017;71(8):968–972. 28378851 10.1038/ejcn.2017.46

[4] Herrera E, Ortega-Senovilla H. Implications of lipids in neonatal body weight and fat mass in gestational diabetic mothers and non-diabetic controls. Curr Diab Rep. 2018;18(2):7. 29399727 10.1007/s11892-018-0978-4

[5] Whalley LJ, Fox HC, Wahle KW, et al. Cognitive aging, childhood intelligence, and the use of food supplements: possible involvement of n-3 fatty acids. Am J Clin Nutr. 2004;80(6):1650–1657. 15585782 10.1093/ajcn/80.6.1650

[6] Koletzko B, Agostoni LE, C, et al. The roles of long-chain polyunsaturated fatty acids in pregnancy, lactation, and infancy: review of current knowledge and consensus recommendations. J Perinat Med. 2008;36:5–14. 18184094 10.1515/JPM.2008.001

[7] Neuringer M, Reisbick S, Janowsky J. The role of n-3 fatty acids in visual and cognitive development: current evidence and methods of assessment. J Pediatr. 1994;125(5):S39–S47. 7965452 10.1016/s0022-3476(06)80735-3

[8] Hanebutt FL, Demmelmair H, Schiessl B, et al. Long-chain polyunsaturated fatty acid (LC-PUFA) transfer across the placenta. Clin Nutr. 2008;27(5):685–693. 18639956 10.1016/j.clnu.2008.05.010

[9] Herrera E. Lipid metabolism in pregnancy and its consequences in the fetus and newborn. ENDO. 2002;19(1):43–55. 10.1385/ENDO:19:1:43 12583601

[10] Man MZ, Hui TY, Schaeffer JE, et al. Regulation of the murine adipocyte fatty acid transporter gene by insulin. Mol Endocrinol. 1996;10:1021–1028. 8843418 10.1210/mend.10.8.8843418

[11] Knipp GT, Liu B, Audus KL, et al. Fatty acid transport regulatory proteins in the developing rat placenta and in trophoblast cell culture models. Placenta. 2000;21(4):367–375. 10833372 10.1053/plac.1999.0484

[12] Bass NM. The cellular intestinal fatty acid-binding proteins: aspects of structure, regulation, and function. Int Rev Cytol. 1988;111:143–184. 3074959 10.1016/s0074-7696(08)61733-7

[13] Mocking RJT, Steijn K, Roos C, et al. Omega-3 fatty acid supplementation for perinatal depression: a meta-analysis clin. J Clin Psychiatry. 2020;81 (5):19r13106. 10.4088/JCP.19r13106 32898343

[14] Olsen SF, Halldorsson TI, Thorne-Lyman AL, et al. Plasma concentrations of long-chain n-3 fatty acids in early and mid-pregnancy and risk of early preterm birth. EBioMedicine. 2018;35:325–333. 30082226 10.1016/j.ebiom.2018.07.009 PMC6156714

[15] Helland IB, Smith L, Saarem K, et al. Maternal supplementation with very-long-chain n-3 fatty acids during pregnancy and lactation augments children’s IQ at 4 years of age. Pediatrics. 2003;111(1):e39–e44. 12509593 10.1542/peds.111.1.e39

[16] Levy JC, Matthews DR, Hermans MP. Correct homeostasis model assessment (HOMA) evaluation uses the computer program. Diabetes Care. 1998;21(12):2191–2192. 9839117 10.2337/diacare.21.12.2191

[17] Folch J, Lees M, Sloane-Stanley GH. A simple method for the isolation and purification of total lipids from animal tissue. J Biol Chem. 1957;226(1):497–509. 13428781

[18] Buckley JD, Howe PR. Long-chain omega-3 polyunsaturated fatty acids may be beneficial for reducing obesity-a review. Nutrients. 2010;2(12):1212–1230. 22254005 10.3390/nu2121212 PMC3257626

[19] Micallef M, Munro I, Phang M, et al. Plasma n-3 polyunsaturated fatty acids are negatively associated with obesity. Br J Nutr. 2009;102(9):1370–1374. 19454127 10.1017/S0007114509382173

[20] Wang L, Manson JE, Rautiainen S, et al. A prospective study of erythrocyte polyunsaturated fatty acid, weight gain, and risk of becoming overweight or obese in middle-aged and older women. Eur J Nutr. 2016;55(2):687–697. 25820817 10.1007/s00394-015-0889-y PMC4587992

[21] Dewailly E, Blanchet C, Lemieux S, et al. n-3 Fatty acids and cardiovascular disease risk factors among the Inuit of Nunavik. Am J Clin Nutr. 2001;74(4):464–473. 11566644 10.1093/ajcn/74.4.464

[22] Munro IA, Garg ML. Dietary supplementation with n-3 PUFA does not promote weight loss when combined with a very low-energy diet. Br J Nutr. 2012;108(8):1466–1474. 22214842 10.1017/S0007114511006817

[23] Demers G, Roy J, Machuca-Parra AI, et al. Fish oil supplementation alleviates metabolic and anxiodepressive effects of diet-induced obesity and associated changes in brain lipid composition in mice. Int J Obes. 2020;44(9):1936–1945. 10.1038/s41366-020-0623-6 32546855

[24] Alvheim AR, Malde MK, Osei-Hyiaman D, et al. Dietary linoleic acid elevates endogenous 2-AG and anandamide and induces obesity. Obesity. 2012;20(10):1984–1994. 22334255 10.1038/oby.2012.38 PMC3458187

[25] Massiera F, Saint-Marc P, Seydoux J, et al. Arachidonic acid and prostacyclin signaling promote adipose tissue development: a human health concern? J Lipid Res. 2003;44(2):271–279. 12576509 10.1194/jlr.M200346-JLR200

[26] Osei-Hyiaman D, DePetrillo M, Pacher P, et al. Endocannabinoid activation at hepatic CB1 receptors stimulates fatty acid synthesis and contributes to diet-induced obesity. J Clin Invest. 2005;115(5):1298–1305. 15864349 10.1172/JCI23057 PMC1087161

[27] Simopoulos AP, DiNicolantonio JJ. The importance of a balanced ω-6 to ω-3 ratio in the prevention and management of obesity. Open Heart. 2016;3(2):e000385. 27843563 10.1136/openhrt-2015-000385 PMC5093368

[28] Makrides M, Best K, Yelland L, et al. A randomized trial of prenatal n − 3 fatty acid supplementation and preterm delivery. N Engl J Med. 2019;381(11):1035–1045. 31509674 10.1056/NEJMoa1816832

[29] Ladfors L, Wiberg N, Katasarou A, et al. Fetal overgrowth in women with type 1 and type 2 diabetes mellitus. PLOS One. 2017;12(11):e0187917. 29121112 10.1371/journal.pone.0187917 PMC5679529

[30] Morrens A, Verhaeghe J, Vanhole C, et al. Risk factors for large-for-gestational age infants in pregnant women with type 1 diabetes. BMC Pregnancy Childbirth. 2016;16(1):162. 27421257 10.1186/s12884-016-0958-0 PMC4946226

[31] Evers IM, de Valk HW, Mol BWJ, et al. Macrosomia despite good glycaemic control in type I diabetic pregnancy: results of a nationwide study in the Netherlands. Diabetologia. 2002;45(11):1484–1489. 12436330 10.1007/s00125-002-0958-7

[32] Ivanisevic M, Delmis K, Herman M, et al. Concentrations of fatty acids among macrosomic neonates delivered by healthy women and women with type 1 diabetes mellitus. Int J Gynecol Obstet. 2020;149(3):309–317. 10.1002/ijgo.13151 32246773

[33] Djelmis J, Ivanisevic M, Desoye G, et al. Higher cord blood levels of fatty acids in pregnant women with type 1 diabetes mellitus. J Clin Endocrinol Metab. 2018;103(7):2620–2629. 29722816 10.1210/jc.2018-00272

[34] Nielsen LR, Rehfeld JF, Pedersen-Bjergaard U, et al. Pregnancy-induced rise in serum C-peptide concentrations in women with type 1 diabetes. Diabetes Care. 2009;32(6):1052–1057. 19244092 10.2337/dc08-1832 PMC2681014

[35] Al MD, van Houwelingen AC, Kester AD, et al. Maternal essential fatty acid patterns during normal pregnancy and their relationship to the neonatal essential fatty acid status. Br J Nutr. 1995;74(1):55–68. 7547829 10.1079/bjn19950106

[36] Food Nutrition Board of the Institute of Medicine. Nutrient Recommendations: Dietary Reference Intakes (DRI). National Institutes of Health, Office of Dietary Supplements. Available online: https://ods.od.nih.gov/. Health_Information/Dietary_Reference_Intakes.aspx

[37] EFSA Panel on Dietetic Products, Nutrition, and Allergies (NDA). Scientific Opinion on Dietary Reference Values for fats, including saturated fatty acids, polyunsaturated fatty acids, monounsaturated fatty acids, trans fatty acids, and cholesterol. EFSA J. 2010;8(3):146.

