# Supplementary material for: Supplementation of EPA and DHA in pregnant women with type 1 diabetes mellitus
Source: Ann Med. 2021 Jul 1;53(1):848–59. doi: 10.1080/07853890.2021.1936151 (PMC8260041; doi:10.1080/07853890.2021.1936151)
Supplement: Supplemental Material [file IANN_A_1936151_SM0040.docx]

Supplemental Table 1. Concentration difference of fatty acids between the third and the first trimester of pregnancy in two study groups

| FAs (mg/L) | Intervention group | Control group group | *P* |
| --- | --- | --- | --- |
| Total fatty acids | 200.6 (131.4 – 345.2) | 198.1 (73.0 – 335.4) | 0.538 |
| Palmitic acid (C16:0) | 51.2 (38.1-58.5) | 48.9 (33.8 – 73.4) | 0.910 |
| Stearic acid (C18:0) | 9.2 (5.1 – 21.8) | 14.1 (1.8 – 23.1) | 0.955 |
| Saturated fatty acid SFA | 76.9 (51.8 – 134.8) | 91.6 (24.6 – 145.0) | 0.485 |
| Palmitoleic acid (C16:1) | 4.0 (13 – 6.8) | 32.0 1.6 – 4.1) | 0.202 |
| Oleic acid (C18:1) | 37.0 (25.2 – 59.5) | 31.2 (10.5 – 69.8) | 0.328 |
| Monounsaturated fatty acid MUFA | 45.2 (29.7 – 66.7) | 37.1 (12.2 – 78.3) | 0.204 |
| Linoleic acid (C18:2) | 53.9 (37.2 – 94.3) | 59.9 (23.4 – 106.7) | 0.671 |
| γ-linolenic acid (C18:3 n-6) | 0.0 (-0.4 – 1.1) | 0.0 (0.0 -1.0) | 0.521 |
| Arachidonic acid (C20:4 n-6) | 8.4 (1.8 – 21.6) | 7.6 (3.3 – 18.1) | 0.781 |
| n-6 polyunsaturated fatty acid (n-6 PUFA) | 68.3 (42.0 -119.0) | 74.0 (25.8 – 129.0) | 0.692 |
| α-linolenic acid (C18:3 n-3) | 1.7 (0.3 -3.2) | 1.4 (0.0 – 2.7) | 0.156 |
| Eicosapentaenoic acid C20:5 n-3 | 1.6 (0.6 -2.7) | 1.4 (0.3 – 3.0) | 0.832 |
| Docosahexaenoic acid C22:6 n-3 | 8.1 (4.2 – 13.1) | 3.4 (1.8 – 6.1) | **<0.001** |
| n-3 PUFA | 10.5 (7.9 -19.7) | 5.7 (2.1 – 11.0) | **<0.001** |

*P* values in bold are significant

Supplemental Table 1 shows the difference in fatty acid concentration between the first and third trimesters. The intervention group had a more significant difference for DHA and n-3 PUFA than the control group.

Supplemental Table 2. Proportion difference of fatty acids between the third and the first trimester of pregnancy

| FAs (%) | Intervention group | Control group | P |
| --- | --- | --- | --- |
| Palmitic acid (C16:0) | 2.9 (1.5 -4.0) | 2.3 (0.3 -3.1) | 0.060 |
| Stearic acid( C18:0) | -1.8 [(-2.3 – (-1.1)] | -1.5 [(-2.0 - (-0.7)] | 0.297 |
| Saturated fatty acid (SFA) | 1.4 (-0.01 -2.5) | 0.7 (-0.8 – 2.2) | 0.328 |
| Palmitoleic acid (C16:1) | 0.3 (-0.1 – 0.5) | 0.03 (-0.3 -0.6) | 0.692 |
| Oleic acid (C18:1) | 0.6 (-1.3 -2.8) | 1.3 (-0.1 -2.4) | 0.461 |
| Monounsaturated fatty acid (MUFA) | 0.8 (-0.5 – 3.0 | 1.1 (-0.6 – 3.2 | 0.539 |
| Linoleic acid (C18:2) | -1.0 (-2.4 – 0.0) | -0.5 (-2.9 – 1.2) | 0.521 |
| γ-linolenic acid (C18:3 n-6) | -0.1 (-0.3 – 0.0) | 0.0 (-0.1 – 0.2) | 0.269 |
| Arachidonic acid (C20:4 n-6) | 0.0 [-0.01 – (-0.4)] | 0.0 (0.0 – 0.0) | 0.161 |
| n-6 polyunsaturated fatty acid (n-6 PUFA) | -4.0 [(-5.2 – (-0.6)] | -2.5 [(-5.0 – (-1.1)] | 0.542 |
| α-linolenic acid (C18:3 n-3) | 0.4 (-0.1 – 0.5) | 0.1 (-0.1 – 0.5) | 0.269 |
| Eicosapentanoic acid (C20:5 n-3) | -0.1 (-0.2 -0.1) | -0.1 (-0.3 – 0.5) | 0.861 |
| Docosahexaenoic acid (C22:6 n-3) | 0.9 (0.2 – 1.3) | 0.01 (-0.3 -0.4) | **<0.001** |
| n-3 polyunsaturated fatty acid (n-3 PUFA) | 1.0 (0.2 – 1.9) | 0.2 (-0.3 - 0.6) | **<0.001** |
| AA/DHA | 2.0 (3.2 – 1.0) | 1.3 (2,4 – 0.5) | **0.048** |

*P* values in bold are significant.

Supplemental Table 2 shows the difference in fatty acid proportions between the first and third trimesters. The intervention group had a more significant difference in DHA, n-3 PUFA, and AA / DHA compared to the control group.

Supplemental Table 3. Proportion of fatty acids in maternal and umbilical vein serum at birth

|  | **Maternal serum** | | | **Umbilical vein serum** | | | **Ratio maternal serum: umbilical vein serum** | | |
| --- | --- | --- | --- | --- | --- | --- | --- | --- | --- |
| **Fatty acids**  **(µg/100 µg of fatty acid)** | Iintervention group (n=50) | Control group (n=50) | *P* | Intervention group (n=50) | Control group (n=50) | *P* | Intervention group (n=42) | Control group (n=42) | *P* |
| Palmitic acid (C16:0) | 29.1  (28.0-29.9) | 29.2  (27.5-30.7) | 0.785 | 30.4  (28.9-31.9) | 31.3  (29.6-32.6) | 0.424 | 0.98  (0.9-1.0) | 0.95  (0.9-1.0) | 0.865 |
| Stearic acid (C18:0) | 7.2  (6.5-7.3) | 7.1  (6.3-7.9) | 0.954 | 11.4  (11.0-12.2) | 11.2  (10.4-11.8) | 0.171 | 0.6  (0.55-0.67) | 0.63  (0.58-0.7) | 0.122 |
| SFAs | 38.0  (37.0-39.2 | 38.6  (37.3-41.2) | 0.190 | 43.9  (42.8-45.3) | 44.9  (43.1-47.8) | 0.050 | 0.87  (0.83-0.89) | 0.87  (0.83-0.91) | 0.631 |
| Palmitoleic acid (C16:1 n-7) | 1.9  (1.4-2.3) | 1.5  (1.1-1.8) | **0.031** | 3.4  (3.0-3.9) | 3.2  (2.7-4.0) | 0.458 | 0.52  (0.4-0.67) | 0.41  (0.32-0.84) | 0.078 |
| Oleic acid (C18:1 n-9) | 19.4  (17.6-21.1) | 19.2  (18.4-21.2) | 0.607 | 14.9  (13.9-16.2) | 16.1  (13.1-17.6) | 0.197 | 1.3  (1.1-1.5) | 1.2  (1.1-1.5 | 0.849 |
| MUFAs | 39.0  (36.8-41.4) | 39.5  (36.3-40.7) | 0.788 | 20.1  (18.7-21.2) | 20.5  (16.5-23.0) | 0.859 | 1.93  (1.85-2.17) | 1.84  (1.60-2.41) | 0.379 |
| α-linolenic acid (C18:3 n-3) | 0.52  (0.4-0.65) | 0.44  (0.0-0.6) | **0.035** | 0.0  (0.0-0.0) | 0.0  (0.0-0.0) | NA |  |  |  |
| Eicosapentanoic acid (C20:5 n-3) | 0.7  (0.5-0.8) | 0.8  (0.5-1.0) | 0.281 | 1.0  (0.0-1.2) | 0.0  (0.0-1.1) | 0.154 | 2.3  (1.9-2.9) |  |  |
| Docosahexaenoic acid (C22:6 n-3) | 2.4  (2.0-3.0) | 1.8  (1.4-2.5) | **0.002** | 3.6  (2.5-4.1) | 2.6  (2.1-3.1) | **0.003** | 0.7  (0.6-0.8) | 0.7  (0.6-0.9) | 0.972 |
| n-3 PUFA | 3.9  (3.3-4.6) | 3.1  (2.5-3.9) | **0.001** | 4.6  (3.8-5.3) | 3.1  (2.4-4.1) | **<0.001** | 0.85  (0.68-0.98) | 0.97  (0.8-1.24) | **0.044** |
| Linoleic acid (C18:2 n-6) | 26.0  (24.3-28.3) | 27.0  (24.6-28.8) | 0.434 | 11.3  (10.0-12.0) | 11.0  (19.3-12.7) | 0.928 | 2.4  (2.1-2.8) | 2.4  (1.9-2.7) | 0.734 |
| γ-linolenic acid (C18:3 n-6) | 0.2  (0.0-0.3) | 0.0  (0.0-0.3) | 0.161 | 0.46  (0.0-0.74) | 0.0  (0.0-0.20) | **<0.001** | 0.31  (0.19-0.45) | 0.36  (0.0-0.53) | 0.523 |
| Arachidonic acid (C20:4 n-6) | 6.1  (5.1-6.9) | 6.2  (5.4-7.7) | 0.312 | 13.2  (12.3-14.6) | 14.4  (12.8-16.6) | **0.020** | 0.44  (0.4.0.52) | 0.45  (0.38-0.52) | 0.737 |
| n-6 PUFA | 34.8  (33.4-36.6) | 35.9  (33.4-37.3) | 0.366 | 30.3  (28.7-31.8) | 31.1  (28.9-33.5) | 0.254 | 1.2  (1.1-1.3) | 1.2  (1.0-1.3) | 0.589 |
| AA/DHA | 2.4  (2.0-3.3) | 3.4  (2.9-4.3) | **0.001** | 3.8  (3.2-4.6) | 5.6  (4.5-6.5) | **<0.001** | 0.7  (0.6-0.8) | 0.7  (0.6-0.8) | 0.574 |
| LA/AA | 4.1  (3.6-5.4) | 4.4  (3.2-4.9) | 0.738 | 0.9  (0.7-1.0 | 0.8  (0.7-0.9) | 0.763 | 5.5  (4.4-6.1) | 4.9  (4.2-6.4) | 0.110 |

*P* values in bold are significant

Supplemental Table 4. HbA1c, GWG, and n-6 PUFAs proportion in

umbilical vein serum of macrosomal and eutrophic neonates

|  | Makrosomal neonates | Eutrophic neonates | *P* |
| --- | --- | --- | --- |
| HbA1c 1st trimester | 7.3±1.1 | 6.6±1.1 | **0.022** |
| HbA1c 2nd trimester | 6.1±5.5 | 5.5±0.6 | **0.001** |
| HbA1c 3rd trimester | 6.6±0.5 | 5.7±0.8 | **<0.001** |
| GWG | 16.6±4.0 | 12.5±4.5 | **0.002** |
| Umbilical vein serum | | | |
| n-6 PUFAs | 28.2  (24.8-29.7) | 31.1  (29.5-32-3) | **0.016** |

Supplemental Table 5. Own evaluation participants about the consumption of olive oil, sea fish and seafood and smoking

|  | Intervention group | Control group | *P* |
| --- | --- | --- | --- |
| Olive oil No/Yes n  (%) | 12/30  (28.6/71.4) | 14/28  (33.3/66.7) | 0.814 |
| Sea fish and seafoods No/Yes n  (%) | 30/12  (75/25) | 26/12  (68.4/31.6) | 0.617 |
| Smoking No/Yes n  (%) | 35/7  (63.3/16.7) | 37/5  (88.1/17.9) | 0.756 |

Supplememtal Table 5 shows participants own evaluation about the consumption of olive oil, sea fish, seafood, and smoking.

There was no significant difference about consumption of olive oil, sea fish, and smoking between intervention and control group.

Supplemental Figure 1. Correlation between the concentration of DHA in maternal vein serum and DHA in umbilical vein serum (r_rho_=0.550: P< 0.001)

A high correlation coefficient (r_rho_ = 0.550; P< 0.001) of DHA concentration between maternal and umbilical venous serum was obtained.

Supplemental Figure 2. Correlation between concentration of AA in maternal vein serum and umbilical vein serum (r_rho_= 0.420: P <0.001)

A high correlation coefficient (r_rho_= 0.420; P <0.001) of AA concentration between maternal and umbilical venous serum was obtained.
